# Crystal structure of tetra­kis­(μ_3_-2-{[1,1-bis­(hy­droxy­meth­yl)-2-oxidoeth­yl]imino­meth­yl}-6-meth­oxy­phenolato)tetra­kis­[aqua­copper(II)]: a redetermination at 200 K

**DOI:** 10.1107/S2056989015017314

**Published:** 2015-09-26

**Authors:** Elena A. Buvaylo, Olga Yu. Vassilyeva, Brian W. Skelton

**Affiliations:** a Department of Inorganic Chemistry, Taras Shevchenko National University of Kyiv, 64/13 Volodymyrska Street, Kyiv 01601, Ukraine; b Centre for Microscopy, Characterisation and Analysis, M313, University of Western Australia, Perth, WA 6009, Australia

**Keywords:** crystal structure, Cu^II^ cubane-type complex, Schiff base ligand, *o*-vanillin, tris­(hy­droxy­meth­yl)amino­methane, hydrogen bonding

## Abstract

The crystal structure of the tetra­nuclear title compound, [Cu_4_(C_12_H_15_NO_5_)_4_(H_2_O)_4_], has been previously reported by Back, Oliveira, Canabarro & Iglesias [*Z. Anorg. Allg. Chem.* (2015), **641**, 941–947], based on room-temperature data. In the previously published structure, no standard uncertainties are recorded for the deprotonated hy­droxy­methyl group and water mol­ecule O atoms coordinating to the metal atom indicating that they were not refined; furthermore, the H atoms of some OH groups and water mol­ecules have not been positioned accurately. Since the current structure was determined at a lower temperature, all atoms, including the H atoms of these hy­droxy groups and the water mol­ecule, have been determined more accurately resulting in improved standard uncertainties in the bond lengths and angles. Diffraction data were collected at 200 K, rather than the more usual 100 K, due to apparent disordering at lower temperatures. In addition, it is now possible to report intra- and inter­molecular O—H⋯O inter­actions. In the title complex molecule, which has crystallographic -4 symmetry, the Cu^II^ ions are coordinated by the tridentate Schiff base ligands and water mol­ecules, forming a tetra­nuclear Cu_4_O_4_ cubane-like core. The Cu^II^ ion adopts a CuNO_5_ elongated octa­hedral environment. The coordination environment of Cu^II^ at 200 K displays a small contraction of the Cu—N/O bonds, compared with the room-temperature structure. In the crystal lattice, the neutral clusters are linked by inter­molecular O—H⋯O hydrogen bonds into a one-dimensional hydrogen-bonding network propagating along the *b* axis.

## Abstract 2

Using a predesigned Schiff base tripodal ligand, a cubane-type tetra­nuclear copper(II) cluster has been synthesized and its structure redetermined at 200 K.

## Chemical context   

During the last few years, we have been exploring the chemistry of transition metal complexes of Schiff base ligands with the aim of preparing heterometallic polynuclear compounds with diverse potential advantages. In these studies, we continued to apply the *direct synthesis of coordination compounds* based on spontaneous self-assembly, in which one of the metals is introduced as a powder (zerovalent state) and oxidized during the synthesis (typically by di­oxy­gen from the air) (Pryma *et al.*, 2003 ▸; Nesterova *et al.*, 2008 ▸; Nesterov *et al.*, 2012 ▸). The main advantage of this approach is the generation of building blocks *in situ*, in one reaction vessel, thus eliminating separate steps in building-block construction. Reactions of a metal powder and another metal salt in air with a solution containing a pre-formed Schiff base ligand have yielded a number of novel Co/Fe and Cu/Fe compounds (Chygorin *et al.*, 2015 ▸; Nesterova *et al.*, 2013 ▸).
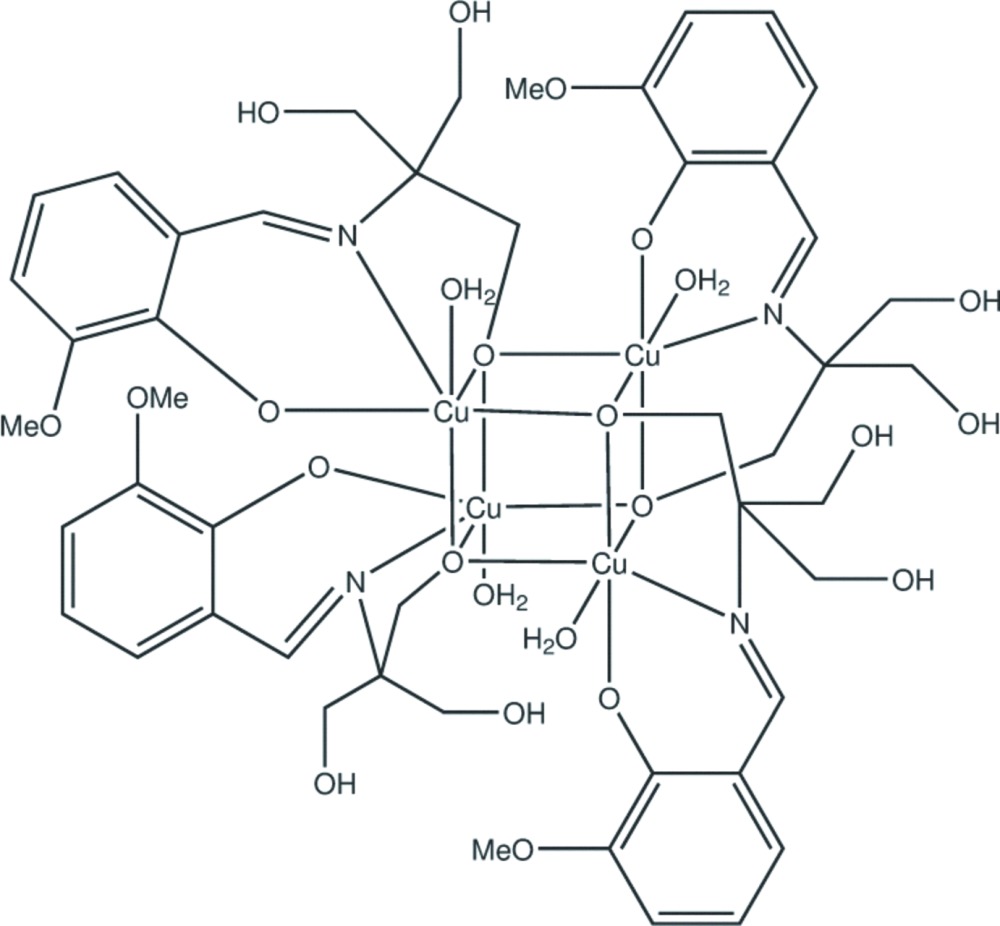


The title compound was prepared in studies of the coordination behavior of the versatile multidentate Schiff base ligand 2-{[(2-hy­droxy-3-meth­oxy­phen­yl)methyl­ene]amino}-2-(hy­droxy­meth­yl)-1,3-propane­diol (H_4_L) (Odabaşoğlu *et al.*, 2003 ▸) which results from the condensation between *o*-vanillin and tris­(hy­droxy­meth­yl)amino­methane. In the syntheses, the condensation reaction was utilized without isolation of the resulting Schiff base. In an attempt to prepare a heterometallic assembly we reacted Cu powder and Zn(CH_3_COO)_2_ with a methanol solution of the Schiff base in a 1:1:2 molar ratio. However, the isolated green microcrystalline product was identified crystallographically to be the tetra­nuclear Cu^II^ Schiff base complex Cu_4_(H_2_L)_4_(H_2_O)_4_ (**1**) of a hetero-cubane type.

The crystal structure of (**1**) has been reported previously at room temperature by Back *et al.* (2015 ▸) (refcode IGOSUU). In that report of the structure, no standard uncertainties are recorded for the oxygen atoms of the deprotonated hy­droxy­methyl group, O2, and the water mol­ecule coordin­ating to the metal atom, O6, indicating that they were not refined. The hydrogen atoms of some OH groups and water mol­ecules have also not been positioned accurately. It is clear from the *checkCIF* output that at least one of the water mol­ecule hydrogen atoms, H6B, and one OH hydrogen atom, H4, are incorrectly positioned. Since the present structure was determined at a lower temperature, all atoms, including these hydrogen atoms, have been determined more accurately, resulting in improved standard uncertainties in the bond lengths and angles.

## Structural commentary   

**Figure 1:**
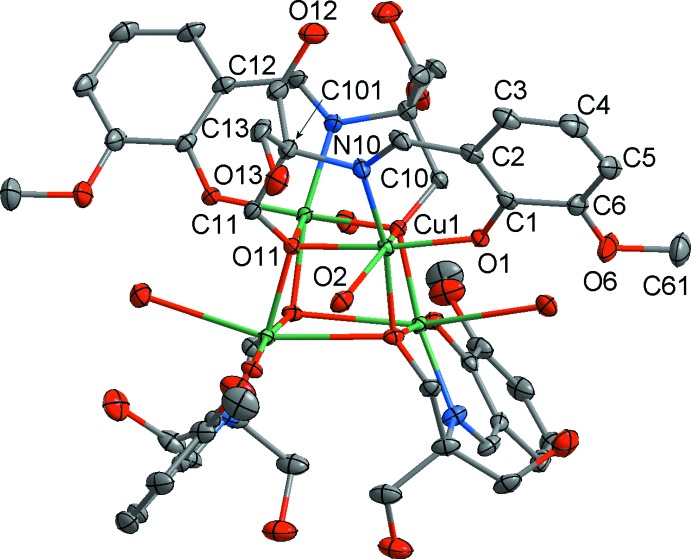
The mol­ecular structure of the title complex, showing the atom-numbering scheme. Non-H atoms are shown with displacement ellipsoids at the 50% probability level. H atoms are not shown.

The neutral [Cu_4_(C_12_H_15_NO_5_)_4_(H_2_O)_4_] mol­ecule of (**1)** has crystallographic 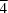 inversion symmetry. The Cu^II^ ions are coordinated by the tridentate Schiff base ligands and water mol­ecules, forming a tetra­nuclear Cu_4_O_4_ cubane-like configuration. The ligand acts in a chelating–bridging mode *via* phenoxo-, alkoxo-O and imine-N atoms. The two hy­droxy­methyl groups remain protonated. The coordination about the Cu^II^ atom is distorted octa­hedral as a result of a significant Jahn–Teller distortion, the two axial distances Cu1—O2 2.738 (5) Å (to the water mol­ecule) and the bridging bond, Cu1—O11 2.547 (4) Å, being significantly longer than the remainder which lie in the range 1.912 (4)–1.968 (3) Å (Fig. 1 ▸, Table 1 ▸). The *trans* angles at the metal atom lie in the range 159.30 (12)–171.70 (15)°, while the *cis* ones vary from 73.02 (12) to 116.70 (16)°. The Cu⋯Cu distances within the Cu_4_O_4_ core are 3.1724 (8) and 3.4474 (8) Å.

There are intra­molecular O2—H2*AO*⋯O13 hydrogen bonds between a hydrogen atom of the water mol­ecule and the oxygen atom of one hy­droxy­methyl group. A further intra­molecular hydrogen bond involves the other hy­droxy­methyl group (O12). Bifurcated inter­molecular hydrogen bonds are also present, involving the remaining hydrogen atom of water mol­ecule and the phenolic and methoxyl oxygen atoms. These hydrogen-bond contacts are of weak-to-moderate strength [2.736 (12)–2.892 (7) Å], Table 2 ▸.

The title compound appears to be a new solvatomorph of the blue copper(II) complex with the same ligand, [Cu_4_(C_12_H_15_NO_5_)_4_(H_2_O)]·3.75CH_3_OH·2H_2_O (refcode SUGKUC; Tabassum & Usman, 2015 ▸). Monoclinic SUGKUC crystallizes in the *P*2_1_/*n* space group and has no crystallographically imposed symmetry. It is also a cubane-type complex but with some of the coordinating water mol­ecules replaced by other solvents. The bond lengths and angles of (**1**) are comparable to those in the Ni^II^ analogue (refcode ZEHGUQ; Guo *et al.*, 2008 ▸) and a Cu^II^ complex with a similar ligand (refcode AFIMUY; Dong *et al.*, 2007 ▸). The ligand of the latter does not have the meth­oxy group and the copper atom is five-coordinate, the structure lacking the coordinating water mol­ecule of (**1**).

## Supra­molecular features   

**Figure 2:**
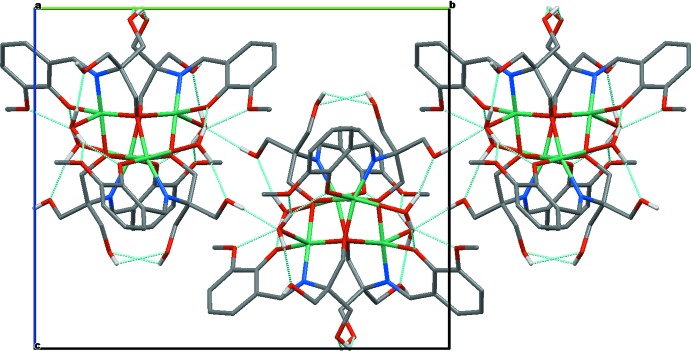
Part of the crystal structure with intra- and inter­molecular hydrogen bonds shown as blue dashed lines. C—H hydrogens have been omitted for clarity.

Inter­actions between [Cu_4_(H_2_ *L*)_4_(H_2_O)_4_] mol­ecules in the crystal lattice are weak, the closest Cu⋯Cu inter-cluster separation exceeds 8.43 Å. The hydrogen on the hy­droxy­methyl group (O13) is involved in an inter­molecular hydrogen bond to the water mol­ecule on the cluster related by a crystallographic twofold axis (Table 2 ▸), forming a hydrogen-bonded polymer propagating along the *b* axis (Fig. 2 ▸). No π–π stacking is observed.

## Database survey   

In the solid state, the H_4_ *L* ligand adopts the keto–amine tautomeric form, with the formal ar­yl–OH H atom relocated to the N atom, and the NH group and phenolic O atom forming a strong intra­molecular N—H⋯O hydrogen bond (Odabaşoğlu *et al.*, 2003 ▸). Crystal structures of about 30 metal complexes of this ligand are found in the Cambridge Database (CSD Version 5.36 with one update; Groom & Allen, 2014 ▸). These comprise five homometallic mononuclear Mn, Ni and Mo complexes, polynuclear Co_2_, V_2_, Cu_4_, Mn_4_, Ni_4_, Ln_9_ and Ln_10_ assemblies and heterometallic 1*s*–3*d* and 3*d*–4*f* clusters of 4–20 nuclearity. The ligand mol­ecules exist in either doubly or triply deprotonated forms and adopt a chelating-bridging mode, forming five- and six-membered rings. Obviously, the H_4_ *L* ligand favours formation of polynuclear paramagnetic clusters due to the presence of the tripodal alcohol functionality. At the same time, the lack of heterometallic structures with two kinds of 3*d* metal supported by H_4_ *L* is also evident. This perhaps explains the failure of the preparation of a Cu/Zn compound in the present study.

## Synthesis and crystallization   

2-Hy­droxy-3-meth­oxy-benzaldehyde (0.30 g, 2 mmol), tris(hy­droxy­meth­yl)amino­methane (0.24 g, 2 mmol), NEt_3_ (0.3 ml, 2 mmol) were added to methanol (20 ml) and stirred magnetically for 30 min. Next copper powder (0.06 g, 1 mmol) and Zn(CH_3_COO)_2_ (0.19 g, 1 mmol) were added to the yellow solution and the mixture was heated to 323 K under stirring until total dissolution of the copper powder was observed (1 h). The resulting green solution was filtered and allowed to stand at room temperature. Dark-green rhombic prisms of the title compound were formed in several days. They were collected by filter-suction, washed with dry Pr^*i*^OH and finally dried *in vacuo* (yield: 59% based on copper).

The IR spectrum of (**1**) in the range 4000–400 cm^−1^ shows all the characteristic Schiff base ligand frequencies: ν(OH), ν(CH) and ν(C=N) at 3400, 3066–2840, and 1604 cm^−1^, respectively. A strong peak at 1628 cm^−1^ that is due to the bending of H_2_O mol­ecule provides evidence of the presence of water in (**1**).

## Refinement   

Crystal data, data collection and structure refinement details are summarized in Table 3 ▸. Diffraction data were collected at 200 K, rather than the more usual 100 K, due to apparent disordering at lower temperatures. Water mol­ecule hydrogen atoms were refined with geometries restrained to ideal values; the OH hydrogen atoms H12 and H13 were refined using a riding model. All hydrogen atoms bound to carbon were included in calculated positions and refined using a riding model with isotropic displacement parameters based on those of the parent atom [C—H = 0.95 Å, *U*_iso_(H) = 1.2*U*_eq_(C) for CH and CH_2_, 1.5*U*_eq_(C) for CH_3_). Anisotropic displacement parameters were employed for the non-hydrogen atoms.

## Supplementary Material

Crystal structure: contains datablock(s) I, global. DOI: 10.1107/S2056989015017314/sj5474sup1.cif

Structure factors: contains datablock(s) I. DOI: 10.1107/S2056989015017314/sj5474Isup2.hkl

Supporting information file. DOI: 10.1107/S2056989015017314/sj5474Isup3.pdf

CCDC reference: 1424781

Additional supporting information:  crystallographic information; 3D view; checkCIF report

## Figures and Tables

**Table 1:** Selected bond lengths ()

Cu1O1	1.912(4)	Cu1O2	2.738(5)
Cu1O11	1.941(4)	Cu1O11^i^	1.968(3)
Cu1N10	1.953(5)	Cu1O11^ii^	2.547(4)

Symmetry codes: (i) 
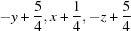
; (ii) 
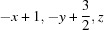
.

**Table 2:** Hydrogen-bond geometry (, )

*D*H*A*	*D*H	H*A*	*D* *A*	*D*H*A*
O12H12O12^ii^	0.84	2.37	2.736(12)	107
O13H13O2^iii^	0.84	1.91	2.700(6)	156
O2H2*AO*O1^iv^	0.93(5)	1.92(4)	2.791(6)	155(8)
O2H2*AO*O6^iv^	0.93(5)	2.23(7)	2.853(7)	124(6)
O2H2*BO*O13	0.96(5)	1.95(3)	2.892(7)	165(6)

Symmetry codes: (ii) 
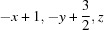
; (iii) 
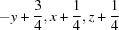
; (iv) 
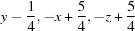
.

**Table 3:** Experimental details

Crystal data	
Chemical formula	[Cu_4_(C_12_H_15_NO_5_)_4_(H_2_O)_4_]
*M*_r_	1339.22
Crystal system, space group	Tetragonal, *I*4_1_/*a*
Temperature (K)	200
*a*, *c* ()	18.7108(3), 15.3800(3)
*V* (^3^)	5384.4(2)
*Z*	4
Radiation type	Mo *K*
(mm^1^)	1.65
Crystal size (mm)	0.39 0.23 0.17

Data collection	
Diffractometer	Oxford Diffraction Xcalibur
Absorption correction	Analytical (*CrysAlis CCD* and *CrysAlis RED*; Agilent, 2013 ▸)
*T*_min_, *T*_max_	0.687, 0.843
No. of measured, independent and observed [*I* > 2(*I*)] reflections	25330, 3247, 2942
*R*_int_	0.044
(sin /)_max_ (^1^)	0.660

Refinement	
*R*[*F* ^2^ > 2(*F* ^2^)], *wR*(*F* ^2^), *S*	0.076, 0.195, 1.12
No. of reflections	3247
No. of parameters	188
No. of restraints	4
H-atom treatment	H atoms treated by a mixture of independent and constrained refinement
_max_, _min_ (e ^3^)	1.58, 0.98

Computer programs: *CrysAlis CCD* (Agilent, 2013 ▸), *SIR92* (Altomare *et al.*, 1994 ▸), *SHELXL2014* (Sheldrick, 2015 ▸), *DIAMOND* (Brandenburg, 1999 ▸) and *WinGX* (Farrugia, 2012 ▸).

## supplementary crystallographic information

### Crystal data

**Table:**

[Cu_4_(C_12_H_15_NO_5_)_4_(H_2_O)_4_]	*D*_x_ = 1.652 Mg m^−^^3^
*M**_r_* = 1339.22	Mo *K*α radiation, λ = 0.71073 Å
Tetragonal, *I*4_1_/*a*	Cell parameters from 7964 reflections
Hall symbol: -I 4ad	θ = 2.8–28.9°
*a* = 18.7108 (3) Å	µ = 1.65 mm^−^^1^
*c* = 15.3800 (3) Å	*T* = 200 K
*V* = 5384.4 (2) Å^3^	Prism, dark green
*Z* = 4	0.39 × 0.23 × 0.17 mm
*F*(000) = 2768	

### Data collection

**Table:**

Oxford Diffraction Xcalibur diffractometer	3247 independent reflections
Radiation source: Enhance (Mo) X-ray Source	2942 reflections with *I* > 2σ(*I*)
Graphite monochromator	*R*_int_ = 0.044
Detector resolution: 16.0009 pixels mm^-1^	θ_max_ = 28.0°, θ_min_ = 2.8°
ω scans	*h* = −23→24
Absorption correction: analytical (*CrysAlis CCD* and *CrysAlis RED*; Agilent, 2013)	*k* = −23→24
*T*_min_ = 0.687, *T*_max_ = 0.843	*l* = −19→20
25330 measured reflections	

### Refinement

**Table:**

Refinement on *F*^2^	4 restraints
Least-squares matrix: full	Hydrogen site location: mixed
*R*[*F*^2^ > 2σ(*F*^2^)] = 0.076	H atoms treated by a mixture of independent and constrained refinement
*wR*(*F*^2^) = 0.195	*w* = 1/[σ^2^(*F*_o_^2^) + (0.0811*P*)^2^ + 52.6317*P*] where *P* = (*F*_o_^2^ + 2*F*_c_^2^)/3
*S* = 1.12	(Δ/σ)_max_ < 0.001
3247 reflections	Δρ_max_ = 1.58 e Å^−^^3^
188 parameters	Δρ_min_ = −0.98 e Å^−^^3^

### Special details

**Table:**

Geometry. All e.s.d.'s (except the e.s.d. in the dihedral angle between two l.s. planes) are estimated using the full covariance matrix. The cell e.s.d.'s are taken into account individually in the estimation of e.s.d.'s in distances, angles and torsion angles; correlations between e.s.d.'s in cell parameters are only used when they are defined by crystal symmetry. An approximate (isotropic) treatment of cell e.s.d.'s is used for estimating e.s.d.'s involving l.s. planes.
Refinement. Diffraction data were collected at 200 K, rather than the more usual 100 K, due to apparent disordering at lower temperatures. Water molecule hydrogen atoms were refined with geometries restrained to ideal values.

### Fractional atomic coordinates and isotropic or equivalent isotropic displacement parameters (Å^2^)

**Table:**

	*x*	*y*	*z*	*U*_iso_*/*U*_eq_	
Cu1	0.47922 (3)	0.66025 (3)	0.69100 (4)	0.0279 (2)	
C1	0.5392 (3)	0.5347 (3)	0.7698 (3)	0.0355 (11)	
O1	0.5448 (2)	0.58332 (19)	0.7095 (2)	0.0350 (8)	
C2	0.4880 (3)	0.5355 (3)	0.8386 (4)	0.0384 (12)	
C3	0.4858 (4)	0.4778 (4)	0.8971 (4)	0.0533 (16)	
H3	0.4506	0.4777	0.9416	0.064*	
C4	0.5322 (5)	0.4225 (4)	0.8922 (5)	0.063 (2)	
H4	0.5285	0.3839	0.9322	0.076*	
C5	0.5845 (5)	0.4218 (3)	0.8297 (5)	0.062 (2)	
H5	0.6179	0.3836	0.8277	0.075*	
C6	0.5887 (4)	0.4770 (3)	0.7690 (4)	0.0531 (16)	
O6	0.6377 (4)	0.4824 (3)	0.7043 (4)	0.0781 (18)	
C61	0.6974 (6)	0.4339 (5)	0.7031 (7)	0.094 (3)	
H61A	0.7184	0.4311	0.7614	0.14*	
H61B	0.7334	0.4512	0.6619	0.14*	
H61C	0.681	0.3863	0.6852	0.14*	
C10	0.4376 (3)	0.5937 (4)	0.8514 (4)	0.0453 (14)	
H10	0.4063	0.5904	0.8999	0.054*	
N10	0.4318 (2)	0.6488 (3)	0.8033 (3)	0.0416 (11)	
C101	0.3781 (4)	0.7071 (4)	0.8247 (4)	0.0492 (15)	
C11	0.3629 (3)	0.7439 (3)	0.7393 (4)	0.0384 (12)	
H11A	0.3232	0.7186	0.7099	0.046*	
H11B	0.3468	0.7933	0.7513	0.046*	
O11	0.42189 (18)	0.7465 (2)	0.6827 (2)	0.0322 (7)	
C12	0.4105 (5)	0.7622 (4)	0.8871 (5)	0.065 (2)	
H12A	0.4533	0.7841	0.8601	0.078*	
H12B	0.3753	0.8006	0.8984	0.078*	
O12	0.4299 (3)	0.7293 (3)	0.9664 (3)	0.0827 (18)	
H12	0.4473	0.76	1.0001	0.124*	
C13	0.3123 (4)	0.6764 (4)	0.8649 (4)	0.0551 (17)	
H13A	0.3232	0.6592	0.9243	0.066*	
H13B	0.2751	0.7139	0.8694	0.066*	
O13	0.2862 (3)	0.6191 (3)	0.8141 (4)	0.0724 (16)	
H13	0.2492	0.6023	0.8374	0.109*	
O2	0.3560 (3)	0.5887 (2)	0.6505 (3)	0.0497 (10)	
H2AO	0.341 (4)	0.618 (4)	0.605 (3)	0.075*	
H2BO	0.326 (4)	0.601 (4)	0.699 (3)	0.075*	

### Atomic displacement parameters (Å^2^)

**Table:**

	*U*^11^	*U*^22^	*U*^33^	*U*^12^	*U*^13^	*U*^23^
Cu1	0.0264 (3)	0.0308 (3)	0.0265 (3)	−0.0006 (2)	−0.0008 (2)	0.0045 (2)
C1	0.044 (3)	0.030 (2)	0.033 (3)	−0.003 (2)	−0.013 (2)	0.0002 (19)
O1	0.0390 (19)	0.0342 (18)	0.0317 (17)	0.0055 (15)	0.0023 (15)	0.0060 (14)
C2	0.039 (3)	0.042 (3)	0.034 (3)	−0.010 (2)	−0.008 (2)	0.008 (2)
C3	0.062 (4)	0.051 (4)	0.046 (3)	−0.017 (3)	−0.009 (3)	0.019 (3)
C4	0.094 (6)	0.042 (3)	0.053 (4)	−0.008 (4)	−0.012 (4)	0.015 (3)
C5	0.101 (6)	0.033 (3)	0.052 (4)	0.015 (3)	−0.012 (4)	0.000 (3)
C6	0.082 (5)	0.033 (3)	0.044 (3)	0.014 (3)	−0.004 (3)	−0.004 (2)
O6	0.105 (4)	0.057 (3)	0.073 (3)	0.044 (3)	0.023 (3)	0.012 (3)
C61	0.101 (7)	0.074 (6)	0.106 (8)	0.046 (5)	0.016 (6)	0.006 (5)
C10	0.033 (3)	0.067 (4)	0.036 (3)	0.000 (3)	0.002 (2)	0.019 (3)
N10	0.033 (2)	0.054 (3)	0.038 (2)	0.011 (2)	0.0041 (19)	0.013 (2)
C101	0.055 (4)	0.054 (4)	0.039 (3)	0.020 (3)	0.009 (3)	0.002 (3)
C11	0.039 (3)	0.040 (3)	0.037 (3)	0.006 (2)	0.009 (2)	−0.005 (2)
O11	0.0298 (17)	0.0392 (19)	0.0274 (17)	0.0051 (14)	−0.0015 (13)	0.0032 (14)
C12	0.095 (6)	0.057 (4)	0.042 (4)	0.026 (4)	0.006 (4)	−0.004 (3)
O12	0.126 (5)	0.081 (4)	0.041 (3)	0.019 (4)	−0.006 (3)	−0.003 (3)
C13	0.060 (4)	0.058 (4)	0.047 (3)	0.015 (3)	0.025 (3)	0.003 (3)
O13	0.049 (3)	0.083 (4)	0.085 (4)	0.002 (3)	0.027 (3)	−0.004 (3)
O2	0.060 (3)	0.037 (2)	0.053 (3)	−0.0002 (19)	0.005 (2)	−0.0109 (19)

### Geometric parameters (Å, º)

**Table:**

Cu1—O1	1.912 (4)	C61—H61C	0.98
Cu1—O11	1.941 (4)	C10—N10	1.273 (7)
Cu1—N10	1.953 (5)	C10—H10	0.95
Cu1—O2	2.738 (5)	N10—C101	1.519 (7)
Cu1—O11^i^	1.968 (3)	C101—C13	1.494 (9)
Cu1—O11^ii^	2.547 (4)	C101—C11	1.510 (8)
C1—O1	1.303 (6)	C101—C12	1.533 (11)
C1—C6	1.422 (8)	C11—O11	1.407 (6)
C1—C2	1.427 (8)	C11—H11A	0.99
C2—C3	1.406 (8)	C11—H11B	0.99
C2—C10	1.455 (9)	O11—Cu1^iii^	1.968 (3)
C3—C4	1.353 (11)	C12—O12	1.414 (9)
C3—H3	0.95	C12—H12A	0.99
C4—C5	1.373 (11)	C12—H12B	0.99
C4—H4	0.95	O12—H12	0.84
C5—C6	1.394 (9)	C13—O13	1.413 (9)
C5—H5	0.95	C13—H13A	0.99
C6—O6	1.356 (9)	C13—H13B	0.99
O6—C61	1.439 (9)	O13—H13	0.84
C61—H61A	0.98	O2—H2AO	0.93 (5)
C61—H61B	0.98	O2—H2BO	0.96 (5)
			
O1—Cu1—O11	171.70 (15)	H61A—C61—H61B	109.5
O1—Cu1—N10	94.41 (17)	O6—C61—H61C	109.5
O11—Cu1—N10	84.16 (17)	H61A—C61—H61C	109.5
N10—Cu1—O2	76.41 (16)	H61B—C61—H61C	109.5
O11—Cu1—O2	85.77 (14)	N10—C10—C2	125.6 (5)
O1—Cu1—O2	101.89 (14)	N10—C10—H10	117.2
O1—Cu1—O11^i^	94.54 (15)	C2—C10—H10	117.2
O11—Cu1—O11^i^	88.41 (16)	C10—N10—C101	120.8 (5)
N10—Cu1—O11^i^	166.34 (18)	C10—N10—Cu1	124.3 (4)
O2—Cu1—O11^ii^	159.30 (12)	C101—N10—Cu1	114.4 (3)
O1—Cu1—O11^i^	94.54 (15)	C13—C101—C11	112.3 (6)
O11—Cu1—O11^i^	88.44 (16)	C13—C101—N10	111.0 (5)
O1—Cu1—O11^ii^	93.29 (13)	C11—C101—N10	105.3 (4)
N10—Cu1—O11^ii^	116.70 (16)	C13—C101—C12	109.1 (6)
O11—Cu1—O11^ii^	80.15 (13)	C11—C101—C12	108.2 (6)
O11^i^—Cu1—O11^ii^	73.02 (12)	N10—C101—C12	110.9 (5)
O2—Cu1—O11^i^	91.64 (15)	O11—C11—C101	113.9 (5)
O2—Cu1—O11^ii^	159.32 (12)	O11—C11—H11A	108.8
O1—C1—C6	118.1 (5)	C101—C11—H11A	108.8
O1—C1—C2	125.1 (5)	O11—C11—H11B	108.8
C6—C1—C2	116.8 (5)	C101—C11—H11B	108.8
C1—O1—Cu1	125.5 (3)	H11A—C11—H11B	107.7
C3—C2—C1	119.2 (6)	C11—O11—Cu1	111.4 (3)
C3—C2—C10	118.0 (6)	C11—O11—Cu1^iii^	121.3 (3)
C1—C2—C10	122.9 (5)	Cu1—O11—Cu1^iii^	108.47 (17)
C4—C3—C2	122.2 (7)	O12—C12—C101	110.4 (6)
C4—C3—H3	118.9	O12—C12—H12A	109.6
C2—C3—H3	118.9	C101—C12—H12A	109.6
C3—C4—C5	120.2 (6)	O12—C12—H12B	109.6
C3—C4—H4	119.9	C101—C12—H12B	109.6
C5—C4—H4	119.9	H12A—C12—H12B	108.1
C4—C5—C6	120.2 (7)	C12—O12—H12	109.5
C4—C5—H5	119.9	O13—C13—C101	110.4 (5)
C6—C5—H5	119.9	O13—C13—H13A	109.6
O6—C6—C5	125.7 (6)	C101—C13—H13A	109.6
O6—C6—C1	113.0 (5)	O13—C13—H13B	109.6
C5—C6—C1	121.3 (7)	C101—C13—H13B	109.6
C6—O6—C61	119.2 (6)	H13A—C13—H13B	108.1
O6—C61—H61A	109.5	C13—O13—H13	109.5
O6—C61—H61B	109.5	H2AO—O2—H2BO	105 (3)

Symmetry codes: (i) −*y*+5/4, *x*+1/4, −*z*+5/4; (ii) −*x*+1, −*y*+3/2, *z*; (iii) *y*−1/4, −*x*+5/4, −*z*+5/4.

### Hydrogen-bond geometry (Å, º)

**Table:**

*D*—H···*A*	*D*—H	H···*A*	*D*···*A*	*D*—H···*A*
O12—H12···O12^ii^	0.84	2.37	2.736 (12)	107
O13—H13···O2^iv^	0.84	1.91	2.700 (6)	156
O2—H2*AO*···O1^iii^	0.93 (5)	1.92 (4)	2.791 (6)	155 (8)
O2—H2*AO*···O6^iii^	0.93 (5)	2.23 (7)	2.853 (7)	124 (6)
O2—H2*BO*···O13	0.96 (5)	1.95 (3)	2.892 (7)	165 (6)

Symmetry codes: (ii) −*x*+1, −*y*+3/2, *z*; (iii) *y*−1/4, −*x*+5/4, −*z*+5/4; (iv) −*y*+3/4, *x*+1/4, *z*+1/4.
