# Supplementary figures and images for: Crystal structure of tetra­kis­(μ3-2-{[1,1-bis­(hy­droxy­meth­yl)-2-oxidoeth­yl]imino­meth­yl}-6-meth­oxy­phenolato)tetra­kis­[aqua­copper(II)]: a redetermination at 200 K
Source: Acta Crystallogr E Crystallogr Commun. 2015 Sep 26;71(Pt 10):1203–6. doi: 10.1107/S2056989015017314 (PMC4647398; doi:10.1107/S2056989015017314)

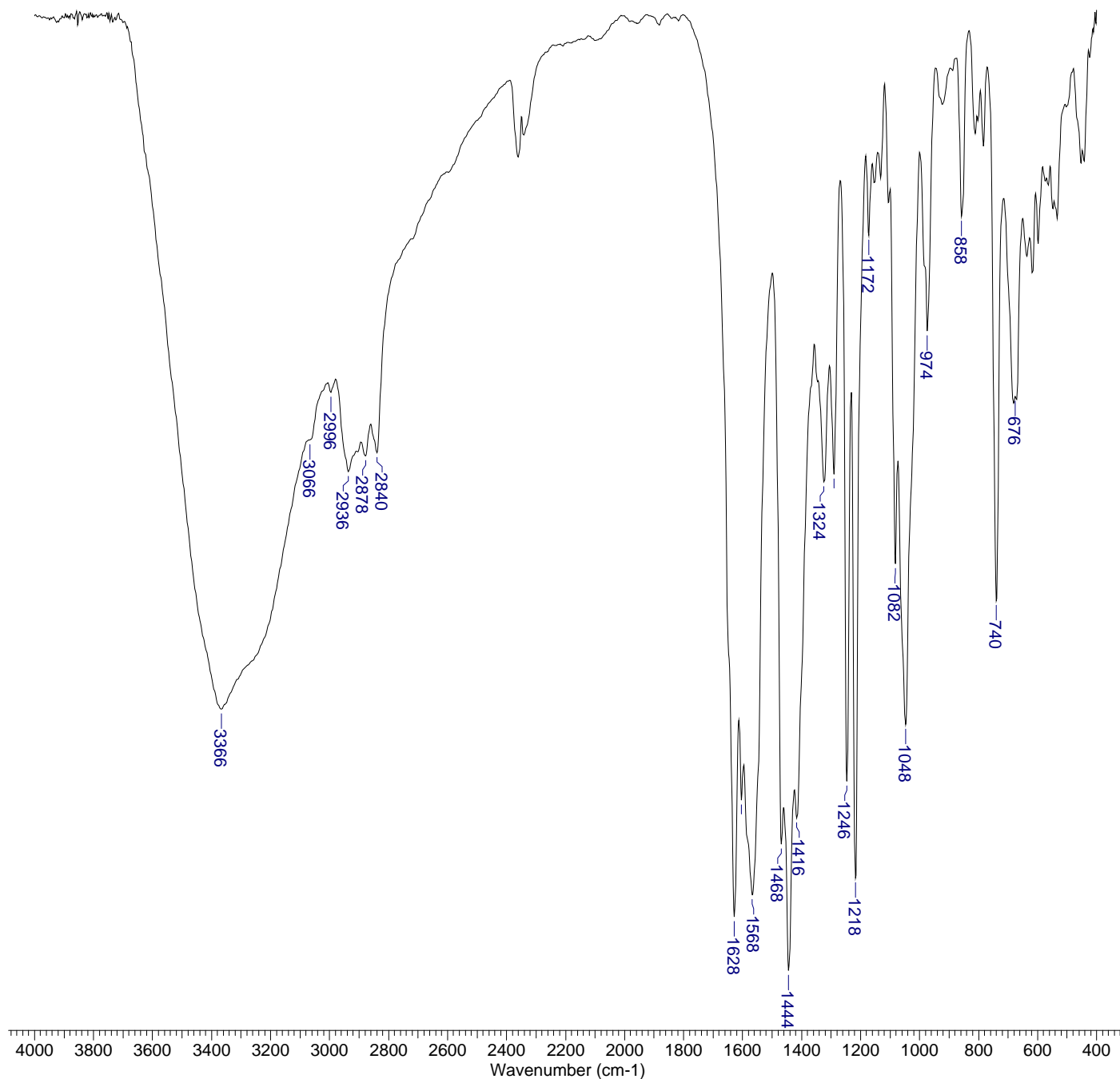

| No | cm-1    | T     | Intensity | No | cm-1    | T     | Intensity |
|----|---------|-------|-----------|----|---------|-------|-----------|
| 1  | 676.00  | 0.001 | M         | 13 | 1444.00 | 0.000 | VS        |
| 2  | 740.00  | 0.001 | S         | 14 | 1468.00 | 0.000 | S         |
| 3  | 858.00  | 0.002 | W         | 15 | 1568.00 | 0.000 | VS        |
| 4  | 974.00  | 0.001 | M         | 16 | 1604.00 | 0.000 | S         |
| 5  | 1048.00 | 0.001 | S         | 17 | 1628.00 | 0.000 | VS        |
| 6  | 1082.00 | 0.001 | M         | 18 | 2840.00 | 0.001 | M         |
| 7  | 1172.00 | 0.002 | W         | 19 | 2878.00 | 0.001 | M         |
| 8  | 1218.00 | 0.000 | VS        | 20 | 2936.00 | 0.001 | M         |
| 9  | 1246.00 | 0.000 | S         | 21 | 2996.00 | 0.001 | M         |
| 10 | 1290.00 | 0.001 | M         | 22 | 3066.00 | 0.001 | M         |
| 11 | 1324.00 | 0.001 | M         | 23 | 3366.00 | 0.001 | S         |
| 12 | 1416.00 | 0.000 | S         |    |         |       |           |

Supplement: Supplementary file 3 [file e-71-01203-Isup3.pdf]
